# Comparative study: how dry heating treatment and annealing influence the multi-structure, physicochemical properties and *in vitro* digestibility of black highland barley starch

**DOI:** 10.3389/fnut.2024.1453424

**Published:** 2024-08-01

**Authors:** Hang Liu, Shanshan Gao, Ge Tian, Si Zhang, Shuang Liu

**Affiliations:** ^1^Shanxi Institute for Functional Food, Shanxi Agricultural University, Taiyuan, China; ^2^School of Food Science and Engineering, Hainan University, Haikou, China; ^3^Hou Ji Laboratory in Shanxi Province, Shanxi Agricultural University, Taiyuan, China

**Keywords:** black highland barley, dry heating treatment, annealing, multi-structures, physicochemical properties, *in vitro* digestibility

## Abstract

In this study, comparative investigation on the effect of dry heating treatment (DHT) and annealing (ANN) on multi-structure, physicochemical properties and *in vitro* digestibility of black highland barley (BHB) starch was done. Results revealed that both DHT and ANN did not affect the “A”-type crystalline pattern and FT-IR spectroscopy of BHB starch, but changed the morphology, raised water absorption capacity and lowered viscosities. Compared to native starch, DHT- and ANN-modified samples had totally opposite alteration trends in amylose content, color characteristics, oil absorption capacity, gelatinization parameters and pasting temperature. These changes were positively related to treatment temperature and time for DHT-modified starches, while which were dependant on treatment duration for ANN-modified starches. Total *in vitro* hydrolysis rate and rapidly digestive starch content in starch markedly raised after DHT, whereas slowly digestive starch and RS levels decreased. Nevertheless, ANN significantly improved the hydrolyzation stability with treatment time prolonging, especially increased RS content and lowered RDS level. Therefore, this study identified both DHT and ANN were effective methods to alter the properties of BHB starch, and more importantly, they had distinguishing influence by different mechanisms, which would remind user to select appropriate means for physical starch modification based on different application purposes.

## 1 Introduction

In China, highland barley (*Hordeum vulgare* L. var. *nudum* Hook. f) is called “Qingke” in Mandarin and “Ne” in Tibetan. It is also regarded as hulless barley or naked barley (1), which is primarily distributed in northwestern and southwestern regions of China, especially on the Qinghai-Tibet Plateau with high altitude of average 4,000 m above sea level (2). Based on the unique geographical conditions including cold, drought, hypoxia

and intense UV radiation, etc., highland barley is rich in various nutrients and bio-active compounds, such as β-glucan, protein, vitamins, phenolics and flavonoids (3), being beneficial to human health with antibacterial, antioxidant even anti-tumorigenic abilities. Meanwhile, highland barley has unique characteristics, like short growth period, wide adaptability, high yield, as well as strong tolerance to cold and drought. Since the fifth century AD, it has been the essential staple food crop for Tibetans (4). In recent decade, studies have proved that consuming highland barley is associated to risk reduction of various chronic diseases, for instance, obesity (5), diabetes (6), hyperlipidemia (7), cardiovascular disease (8), and colonic cancer (9). Thus, as a promising economic crop, highland barley has gained increasing research attention worldwidely.

The hundreds of highland barley varieties can be classified by grain shapes and coat color (10), among which colored highland barley is the precious germplasm resource. Particularly, as one of the most widely cultivated and consumed varieties in Qinghai-Tibet Plateau, black highland barley (BHB) has much higher content of phenolic compounds, dietary fiber, β-glucan and resistant starch than wheat. It is endowed with more potential to regulate cholesterol and postprandial blood glucose levels (11). The dominant single constituent in BHB grains is starch, accounting for 56%−75% of kernel dry weight. The amylose content of highland barley varies from 0 to 40% among different varieties (2). These make highland barley become an important starch resource for food industrial. However, compared to other starches, highland barley starch with longer amylopectin chains has higher gelatinization parameters and stronger freeze-thaw stability (12), which significantly affects the processing property, eating quality and functionality of highland barley products. Thus, it is in urgent need to modify native highland barley starch to further extend food and industrial applications. Recently, some physical technologies have been applied to highland barley starch modification, including dry heating treatment (DHT) (2), microwave irradiation (13), heat moisture treatment (HMT) (14) and roasting (15), but the underling mechanism of physical modification on BHB starch is relatively deficient. The HMT raised amylose content even promoted V-type structure formation in highland barley starch (14), and microwave increased its swelling power and gelatinization properties by disrupting crystalline region (13), whereas the swelling power, viscosity and solubility were decreased by roasting (15). Our previous study revealed the DHT significantly altered the structure and physicochemical properties of blue highland barley starch, especially the RS content (2). However, few comparative studies between different physical modification methods on BHB starch has been done.

Annealing (ANN) is an eco-friendly technology, and DHT is also considered as a “green” method. Compared to chemical treatments, both DHT and ANN are simplicity, safety and low cost (16), and have gained long-standing attention for application in starch modification without destroying the granular structure (2). The ANN is always conducted under excess moisture level and below onset gelatinization temperature of starch (17), while no water involves during the DHT. Thus, the modification mechanisms for DHT and ANN may be totally different. However, to our best knowledge, no comparative research on BHB starch modification by DHT and ANN has been done. Therefore, in this study, ANN treatment under different times and DHT at various temperatures with different durations were applied on BHB starch. Their effects on multi-structure, physicochemical properties and *in vitro* digestibility were systematically unveiled. The obtained results will not only enrich the theoretical basis of BHB modification, but also help promoting future application of BHB in food industry.

## 2 Materials and methods

### 2.1 Materials

The black highland barley (BHB) grains (Kunlun 17#) with 13.8% moisture level were purchased from Qinghai Xinning Biotechnology Co., Ltd. Standard α-amylase from porcine pancreas (A3176, 16 U/mg, Solid), pepsin (P7125; ≥ 400 U/mg) and amyloglucosidase from *Aspergillus niger* (A9913, 100,000 U/g) were purchased from Sigma-Aldrich Chemical Co. (St. Louis, MO, USA). The glucose oxidase-peroxidase (GOPOD) assay kit (K-GLUC) was purchased from Megazyme International Ireland Ltd., (Bray, Ireland). The other chemicals were of analytical grades.

### 2.2 Starch extraction

The extraction of BHB starch was carried out according to our previously published literature (2). The obtained sample was native BHB starch (NBHB) that would be preserved in the fridge at −20°C for future research.

### 2.3 Modification of starch

#### 2.3.1 Dry heating treatment

The NBHB [30 g (dry basis, db)] was evenly distributed to a heat-proof dish in a thin layer (~1 mm). The dish was covered with aluminum foil to avoid loss of material. Then, the sample dishes were heated in a constant temperature convection oven (DHG-9203A, Shanghai Jing Hong Laboratory Instrument Co., Ltd., Shanghai, China) at 150 and 180°C for 2 or 4 h, respectively (2). According to treatment conditions, the samples were labeled as BHB150-2, BHB150-4, BHB180-2, and BHB180-4.

#### 2.3.2 Annealing treatment

The annealing (ANN) was performed on the basis of the procedure reported by Liu et al. (18). Disperse NBHB into distilled water at a ratio of 1:4 (w/v) to make a slurry. It was sealed in a container to equilibrate overnight at 4°C. Subsequently, the containers were incubated at 50°C in the oven for 24, 48, and 72 h, respectively. After cooling to room temperature, the samples were air-dried at 40°C for 12 h after centrifuging. The obtained starch samples were referred to as BHB24, BHB48, and BHB72 according to treatment time, respectively.

### 2.4 Scanning electron microscopy

The morphology of different samples was observed using by a scanning electron microscope (SEM; S3400II, Hitachi Ltd., Tokyo, Japan). The sample was pasted on a double-sided adhesive tape, which was stuck on a metal sample stub. After coating with 20 nm of gold under vacuum, starch samples were observed at an acceleration potential of 20 kV.

### 2.5 Starch crystalline structure

The crystalline pattern of samples was determined with an X-ray diffractometer (XRD, D/MAX 2,500 V, Rigaku Corporation, Japan). The scanning angle (2θ) was programmed from 5 to 60° under 40 kV at 30 mA current, and scanning rate was 4°/min. The Jade software (6.0, OriginLab Corporation, USA) was applied to calculate relative crystallinity (RC, %).

### 2.6 Fourier transform-infrared spectroscopy

A Vertex 70 spectrometer (Bruker Co., Ltd., Ettlingen, Germany) was utilized to record the Fourier transform-infrared spectroscopy (FT-IR) spectra of starches at room temperature. Samples were dried at 40°C in the oven for at least 3 days. The dried sample was mixed with KBr powder at a ratio of 1:150 (sample: KBr, w/w), and the mixture was then ground and compressed into thin pellets. The absorbance of different samples was recorded at the wavenumber from 400 to 4,000 cm^−1^. The ratio of absorbance at 1,047 and 1,022 cm^−1^ (*R*_1, 047/1, 022_) was used to evaluate the short-range ordered structure of starch.

### 2.7 Alkaline water retention and amylose content

The Alkaline water retention (AWR) of native and modified starches was determined according to the method of Liu et al. (19). Sample (1.0 g, db) was transferred into a tube and weighed (W_1_). Subsequently, 0.1 M NaHCO_3_ (5 ml) was added and mixed for 30 s. The mixture was allowed to stand for 20 min under 30 ± 2°C. After incubation, the tube was centrifuged (35 × *g*, 15 min) and drained for 10 min at an angle 10–15° with respect to horizontal. The tube with contents was weighed (W_2_) again. The AWR was calculated as follows,


$$\begin{array}{l} \text{AWR (g/g) of sample}=\;{\text{W}}_{\text{2}}-{\text{W}}_{\text{1}}. \end{array}$$

The amylose content (AMC) was investigated by an iodine-binding procedure reported by Juliano et al. (20). In brief, the defatted starch (100 mg, db) and 1 M NaOH were equilibrated in a flask for 24 h at room temperature. The solution volume was made up to 100 ml with distilled water and vigorously mixed. Starch dispersion (5 ml), 1 M acetic acid and 2 ml of iodine solution were mixed and made up to 100 ml again with distilled water. After 20 min, the absorbance of the mixture was determined at 620 nm.

### 2.8 Color analysis

Color parameters of starches were determined using a colorimeter (CS-821N, Hangzhou CHNSpec Technology Co., Ltd., Hangzhou, China). The system expresses the results in terms of L^*^, a^*^, and b^*^ values, where L^*^ represents lightness; a^*^ represents the green/red components; and b^*^ is the yellow/blue opposition.

### 2.9 Oil and water absorption capacities

The oil and water absorption capacities were determined using the method reported by Liu et al. (19). Starch sample and distill water (or peanut oil) were mixed in a 50 ml centrifuge tube at a ratio of 1:4. During a 30-min incubation at 30°C, the tubes were powerfully stirred every 5 min. Subsequently, the tubes were centrifuged at 7,300 × *g* for 15 min. The volume of decanted supernatant fluid and water (or oil) retained per gram of different samples were calculated.

### 2.10 Solubility and swelling power

The solubility and SP of different starches were detected based on the method reported by Liu et al. (19). Sample (50 mg, db) was loaded directly into a centrifuge tube, weighted (W_1_), and added distilled water (5 ml). Subsequently, the samples were incubated in a shaking water bath for 30 min at 50, 60, 70, 80, and 90°C, respectively. The tubes were centrifuged at 657 × *g* for 15 min after cooling to room temperature. The supernatant was carefully decanted, and the resulting precipitate was weighed (W_2_). Solubility and SP of sample (per 100 g on db) were calculated with following equations:


$$\begin{array}{l} \text{Solubility}=\frac{\text{the weight of dried supernatant}}{\text{weight of sample}}\\ \text{ SP}=\frac{{\text{W}}_{\text{2}}\text{-}{\text{W}}_{\text{1}}}{\text{weight of sample}}. \end{array}$$

### 2.11 Differential scanning calorimetry

Sample gelatinization characteristics were determined using a differential scanning calorimeter (Q2000, TA Instruments, New Castle, DE, USA). The sample (3 mg, db) with 9 ml distilled water, was sealed in a sample pan to equilibrate at room temperature overnight. Then sample pans were heated from 30 to 120°C at 10°C/min with an empty pan as control. The onset temperature (*T*_o_), peak temperature (*T*_p_), conclusion temperature (*T*_c_), and gelatinization enthalpy (Δ*H*) were tested.

### 2.12 Pasting properties analysis

Starch sample (3 g, db) was mixed evenly with distill water (25 g) in an aluminum sample canister. The pasting properties were evaluated by using a Rapid Visco Analyzer (RVA-4, Newport Scientific Co., Ltd., Warriewood, NSW, Australia). Briefly, the testing program was set at 50°C for 1 min, ramped to 95°C in 3.7 min, held at 95°C for 2.5 min, cooled to 50°C in 3.8 min, and finally held at 50°C for 2 min. The peak viscosity (PV), breakdown viscosity (BD), setback viscosity (SB), pasting temperature (PT), and final viscosity (FV) were tested.

### 2.13 *In vitro* digestibility

#### 2.13.1 *In vitro* digestion of starch

The previously published method of Liu et al. (21) was utilized to analyze the *in vitro* digestibility of native and modified starches. Starch (50 mg, db) was put into a 50 ml flask with 5 ml of distilled water. After gelatinization and cooling, 10 ml of HCl-KCl buffer (0.05 M, pH l.5) and 0.2 ml of the pepsin solution were added to each flask. It was incubated in a shaking water bath at 40°C for 60 min. Subsequently, the total sample volume was adjusted to 25 ml with sodium acetate buffer (0.5 M, pH 6.9). Amylase solution (5 ml, 2.6 UI) was added and incubated in a shaking water bath at 37°C for 3 h. Aliquots (1 ml) were obtained from each flask every 10 min during the first 30 min of hydrolysis and every 30 min during the remaining 2.5 h of hydrolysis. The obtained samples were transferred into dry centrifuge tubes, which were immediately placed in boiling water to inactivate the amylase. After cooling, sodium acetate buffer (0.4 M, pH 4.75, 3 ml) and amyloglucosidase (60 μl) were added to each tube. The tubes were then shaken at 60°C for 45 min. The GOPOD kit was used to determine the glucose content of samples, and the starch amount was calculated by multiplying the glucose content by 0.9. The digestion rate was expressed as the percentage of total starch hydrolyzed after different periods.

#### 2.13.2 Resistant starch content

The level of rapid digestible starch (RDS), slow digestible starch (SDS), and resistant starch (RS) was calculated according to the hydrolysis curve. RDS is the hydrated starch in the first 20 min, and SDS is the starch hydrated during 20–120 min. The RS is the remained starch after 180 min.

### 2.14 Statistical analysis

The mean value and standard deviations of data were obtained by triplicate measurements, then statistically analyzed by one-way analysis of variance (ANOVA) with SPSS version 20.0 software (SSPS Inc. Chicago, IL, USA). The differences among the samples were determined using the least significant difference (LSD) test. Statistical significance was set at a level of *p* < 0.05. The principal component analysis (PCA) was performed with Minitab version 17 (Minitab Inc., USA).

## 3 Results and discussion

### 3.1 Morphological properties

**Figure 1:**
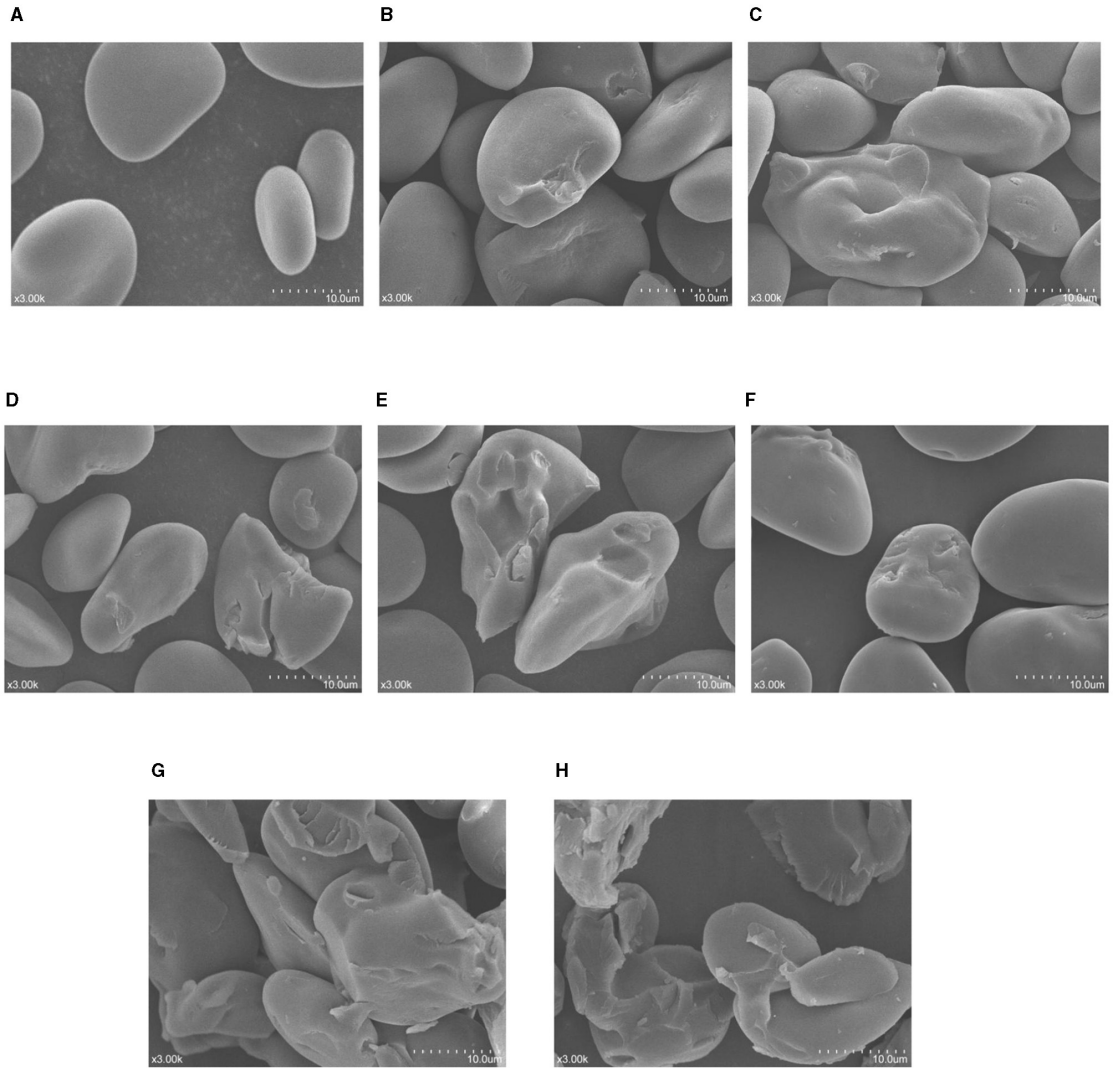
SEM photos (3 K × ) of native and modified samples. **(A)** NBHB; **(B)** BHB150-2; **(C)** BHB150-4; **(D)** BHB180-2; **(E)** BHB180-4; **(F)** BHB24; **(G)** BHB48; **(H)** BHB72.

The SEM photos of native and modified samples are shown in Figure 1. The NBHB granules had smooth surface without any fissures, and the shape of granules was irregular round, oval and polygon (Figure 1A). Some potholes appeared on the surface of BHB150-2 granules following DHT (Figure 1B), and more cavities were observed on the BHB150-4 granules (Figure 1C). Compared to above-mentioned starches, the surface of BHB180-2 granules was obviously eroded, and some larger, deeper pits even holes were found in the hilum (Figure 1D). Besides, more cracks, fissures and holes developed on the BHB180-4 granules (Figure 1E). Compared to that of NBHB, as shown in Figure 1F, some pits were observed on the surface of BHB24 granules, which developed much more and deeper fissures on granules of BHB48 (Figure 1G). Furthermore, the granular structure of BHB72 samples was significantly altered even collapsed (Figure 1H). These results showed the effect of DHT on morphology was positively correlated to treatment temperature and duration, and the influence of ANN was dependant on the processing time. However, these present treatment conditions for both DHT and ANN were insufficient to influence the integrity of NBHB. Similar changes on blue highland, water chestnut and dioscorea starches induced by DHT were reported as well (2, 22, 23).

As the core part, hilum is a ring structure composed of amylose and amylopectin, which grows outward forming the starch granule (2). Due to containing major quantity of moisture, the hilum is soft and more vulnerable to be eroded by heat. This explained the main morphological change and damage in NBHB during DHT happened from hilum. The high temperature resulted in reorganization of amylose-amylopectin and movement of starch molecules, which further contributed to pits, potholes even cracks on the granules (24, 25). Unlike DHT, the main reason for the changes in morphology of NBHB by ANN was the formation of more compact amorphous regions, which induced by the recombination of amylose and amylopectin chains (19). This led to the cavities and holes on the granule surface. Furthermore, the long-time heating of ANN also probably contributed to rearrange the central molecules in starch granules for generating more cracks (26).

### 3.2 Pattern of XRD and relative crystallinity

**Figure 2:**
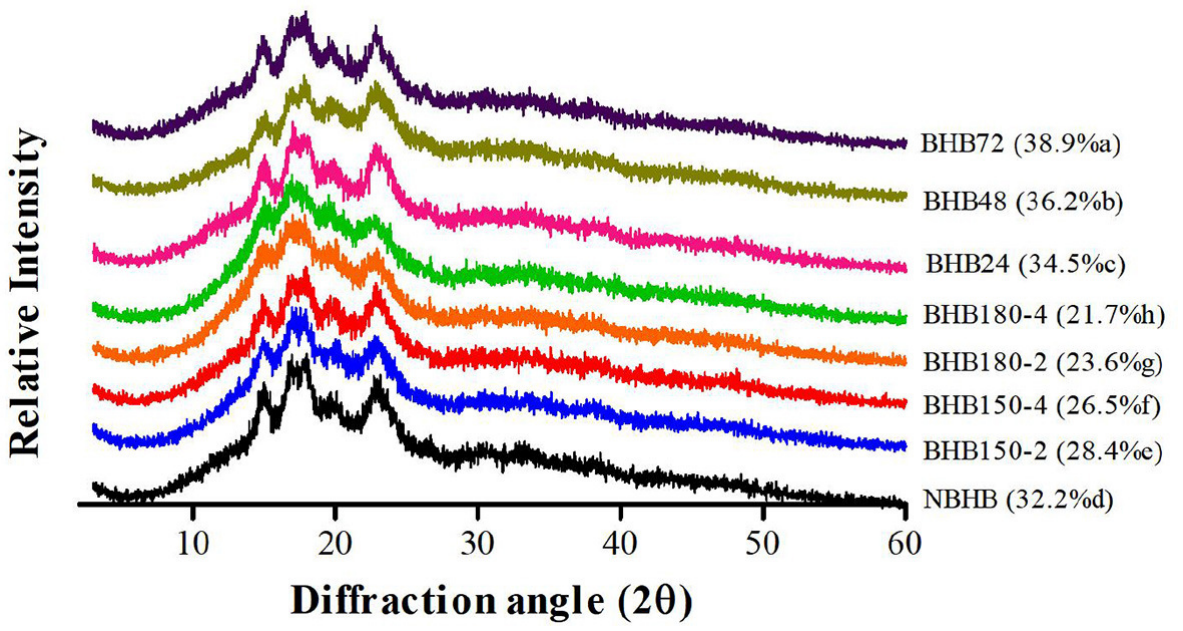
The XRD pattern and relative crystallinity (in parenthesis) of different starches.

The XRD pattern and RC of native and modified starches are presented in Figure 2. The NBHB had a typical “A”-type crystalline pattern with diffraction peaks at 2θ angles of 15.06°, 17.30°, 18.08° and 23.16°. Similar diffraction angles of both DHT- and ANN-modified samples were also detected but with lower diffraction intensity. As shown in Figure 2, this decrease in intensity was positively related to treatment conditions among DHT-modified samples, and correlated to time duration for ANN-modified samples. These results demonstrated that DHT and ANN rarely affected the original “A”-type crystalline pattern of NBHB, and changes in granules might primarily occur in the amorphous region (24). The findings in present study were in agreement with previous researches where the DHT was applied to rice starch (27) and when ANN treatment was used for common buckwheat starch (19).

The RC of NBHB was 32.2%, which significantly decreased after DHT, ranging from 28.4 to 21.7% with the order of BHB150-2 > BHB150-4 > BHB180-2 > BHB180-4. This decrement in RC was dependent on DHT temperature and duration. It might be ascribed to degradation of the crystalline region, partial gelatinization of granules, and interaction of double helices during DHT (28, 29). However, following ANN, the pronouncedly increased RC was observed and positively related to treatment times. The increased interaction among starch chains might enhance new crystal structure formation during ANN, causing the increase in crystallinity (30). This showed that ANN treatment promoted the perfection of crystallites in NBHB.

### 3.3 FT-IR analysis

**Figure 3:**
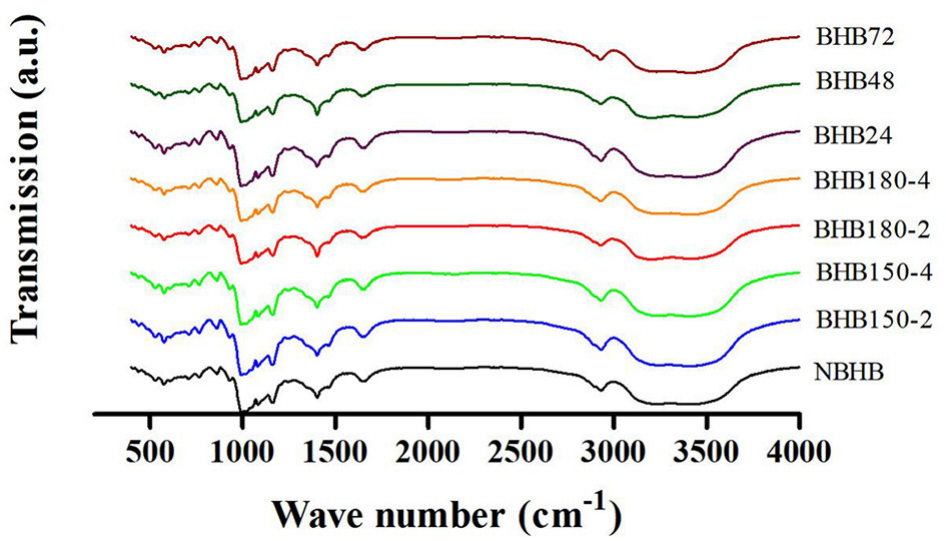
FT-IR spectra of native and modified starch samples.

Generally, FT-IR spectroscopy represents the changes in short-range order of starch (25). The FT-IR spectroscopy of native and modified samples from 400 to 4,000 cm^−1^ is shown in Figure 3, and the absorbance ratio of 1,047/1,022 cm^−1^ (*R*_1047/1022_) is presented in Table 1. Compared to NBHB, all modified samples had similar absorption peaks, suggesting neither new chemical groups were formed nor the existing chemical groups were destroyed during both DHT and ANN with current conditions. The FT-IR bands at 1,047 and 1,022 cm^−1^ indicate the content of crystalline and amorphous structure in starch, respectively. The absorbance ratio of them shows relative level of short-range ordered structure in starch, associated with the crystal and amorphous lamellae density of the starch granule (31).

**Table 1:** Color parameters and FTIR 1,047/1,022 ratio of different samples.

**Samples**	*L* ^*^	*a* ^*^	*b* ^*^	***R*_1, 047/1, 022_**
NBHB	96.20 ± 0.42a	0.14 ± 0.16d	1.70 ± 0.35f	0.95 ± 0.13d
BHB150-2	94.40 ± 0.17b	0.16 ± 0.01d	3.90 ± 0.03d	0.94 ± 0.25d
BHB150-4	93.30 ± 0.29c	0.21 ± 0.02c	5.50 ± 0.05c	0.93 ± 0.34e
BHB180-2	91.20 ± 0.39d	0.87 ± 0.03a	10.80 ± 0.10b	0.92 ± 0.37f
BHB180-4	89.60 ± 0.49e	0.88 ± 0.12a	12.70 ± 0.23a	0.91 ± 0.63g
BHB24	96.10 ± 0.21a	0.14 ± 0.06d	1.80 ± 0.06f	0.97 ± 0.23c
BHB48	95.90 ± 0.36a	0.15 ± 0.12d	1.90 ± 0.21f	0.99 ± 0.08b
BHB72	95.70 ± 0.18a	0.15 ± 0.07d	2.00 ± 0.11e	1.02 ± 0.15a

Means of triplicate determination ± SD with the different letter in a column within each property are significantly different (p <0.05). R_1, 047/1, 022_, the ratio of FTIR value at wave 1,047 and 1,022.

Compared to that of NBHB, the *R*_1, 047/1, 022_ value of DHT-modified starches significantly decreased, which was attributed to the breakdown of original hydrogen bonds in granules, followed by dissociation of double helices in crystalline region (2). Meanwhile, this decreasing trend was positively related to DHT temperature and duration. This identified long treatment time and high temperature during DHT decreased the short-range molecular order in crystalline area of NBHB. Oppositely, the *R*_1, 047/1, 022_ value had been raised after ANN, and this presented that ANN enhanced the short-range order of ANN-modified samples. These results were highly consistent with the XRD results in Section 3.2. Similar results had been reported when mung bean (32), red adzuki bean (33) and sweet potato starches (24) were treated by DHT, and when potato and pea were modified with ANN (31).

### 3.4 Color analysis

The color of starch is vital for food or industrial applications (34). The influence of DHT and ANN on color parameters of NBHB is presented in Table 1. The DHT significantly decreased the L^*^ value from 96.20 (NBHB) to 89.60 (BHB180-4), while increased the a^*^ value from 0.14 (NBHB) to 0.88 (BHB180-4) and b^*^ value from 1.70 (NBHB) to 12.70 (BHB180-4). These changes positively depended on treatment temperature and duration of DHT. However, the L^*^, a^*^, and b^*^ values of ANN-modified starches were not significantly different from those of NBHB, except the b^*^ value of BHB72.

The significant reduction in L^*^ value after DHT showed the NBHB turned darker, which was related to the increase in temperature. The increase in a^*^ value, which is relevant to redness characteristics, indicated more browning in modified samples was induced by DHT, probably due to caramelization reactions (2). Additionally, the rise in b^*^ value of DHT-modified samples suggested that the greenness characteristics of NBHB gradually increased by DHT. Compared to those of NBHB, the color characteristics of ANN-modified starches had not been significantly altered, which was due to low temperature and higher moisture level during ANN. This was in agreement with previous results found by Devi and Sit (35). Similar effect of DHT on color properties of modified potato, sweet potato and taro starches as well as whole-grain barley had also been reported (36). Thus, the DHT-modified starches could be applied to darker products, while ANN-modified starches for food with lighter color.

### 3.5 Oil and water absorption analysis

The oil and water absorption capacities of different samples are shown in Table 2. Compared to NBHB, the DHT-modified starches had significantly higher oil and water absorption capacities. This increment was positively related to DHT temperature and duration, and BHB180-4 had highest water absorption capacity (1.98) and oil absorption capacity (2.11). The ANN treatment also remarkably increased the water absorption capacity of NBHB, while decreased the oil absorption capacity. These changes positively depended on ANN treatment time, with BHB72 having highest water absorption capacity (2.20) and lowest oil absorption capacity (1.14). Similar findings had been reported on cassava starch altered by DHT (36) and tartary buckwheat starch treated by ANN (19).

**Table 2:** AMC, AWR, oil and water absorption capacity of different samples.

**Parameters**	**Samples**							
	**NBHB**	**BHB150-2**	**BHB150-4**	**BHB180-2**	**BHB180-4**	**BHB24**	**BHB48**	**BHB72**
AMC (%)	27.57 ± 0.2d	17.19 ± 0.4e	14.74 ± 0.4f	11.30 ± 0.8g	5.39 ± 0.4d	28.74 ± 0.3c	30.50 ± 0.2b	33.97 ± 0.4a
AWR (g/g)	0.90 ± 0.1f	0.99 ± 0.2e	1.20 ± 0.5d	1.48 ± 0.1c	1.97 ± 0.4a	1.24 ± 0.3d	1.50 ± 0.1c	1.74 ± 0.1b
Water absorption capacity (g/g)	1.38 ± 0.1f	1.58 ± 0.3e	1.77 ± 0.2de	1.81 ± 0.2cd	1.98 ± 0.3bc	1.99 ± 0.5bc	2.06 ± 0.2ab	2.20 ± 0.1a
Oil absorption capacity (g/g)	1.53 ± 0.1e	1.57 ± 0.1d	1.78 ± 0.2c	1.86 ± 0.2b	2.11 ± 1.1a	1.50 ± 0.2e	1.31 ± 0.6f	1.14 ± 0.1g

Means of triplicate determination ± SD with the different letter in a row within each property are significantly different (p < 0.05).

AMC, amylose content; AWR, alkaline water retention.

These results showed there were stronger interaction between hydroxyl and water molecules in DHT-modified starches than that in NBHB, which significantly increased the hydrophilic tendency and induced higher water absorption capacity. Generally, the amorphous region in starch has a higher water absorption capacity than crystalline region. Thus, the results reflected the degree of amorphousness in DHT-modified starch granules was raised, which was in accordance with the results in Section 3.2. Meanwhile, the increase in oil absorption capacity by DHT had been previously found on cassava and wheat starches (37, 38), suggesting DHT-modified starches had potential to be utilized into energy-controlled food. Unlike DHT, some hydrogen bonds between the amorphous and crystalline regions were broken during ANN, inducing the expansion of amorphous region. This would enhance the hydrophilic tendency to increase water absorption capacity of ANN-modified samples. The ANN-modified tartary buckwheat and sorghum starches had shown similar changes in water and oil absorption capacities (19). Moreover, adding modified starches into dough with increased water absorption capacity could improve the color, volume and textural properties of bread (39). This identified applying both DHT- and ANN-modified BHB starches into food products could promote their sensory characteristics.

### 3.6 AWR and AMC analysis

The AMC and AWR of native and modified samples are shown in Table 2. In comparison to that of NBHB, the AMC of DHT-modified starches significantly decreased by 10.38% (BHB150-2), 12.83% (BHB150-4), 16.27% (BHB180-2) and 22.18% (BHB180-4), whereas remarkably increased by 1.17% (BHB24), 2.93% (BHB48) and 6.40% (BHB72) with treatment time raising under ANN, respectively. This decrease in AMC post DHT was positively related to treatment temperature and duration, which was ascribed to the fragmentation of long chains into shorter chains (40). Similar results had also been found in DHT-modified chestnut and cassava starches from previous studies (37, 41). The degradation of amylopectin during ANN was the main reason for increasing AMC. Apart from that, the interactions among starch chains in granules altered the mobility of amorphous and crystalline regions, further resulting in higher iodine-binding capacity of annealed samples (42). The decreased amylose leaching was another factor for the increased AMC of ANN-modified starches (16).

The AWR is an important parameter of starch for processing and application, particularly related to cookie diameter (19). Generally, the cookie diameter is on behalf of the spread potential of a cookie, therefore which can be predicted by the AWR of starch (43). Compared to NBHB, all modified samples had higher AWR levels, which increased with DHT duration and temperature as well as ANN treatment time respectively, and the BHB180-4 had the highest value (1.97). The enhanced surface area (Figure 1) and significantly increased water absorption capacity might jointly account for the markedly raised AWR of the modified starches. Therefore, the utilization of both DHT- and ANN-modified BHB starches will result in a larger cookie diameter compared to that with NBHB. These results suggested the modified BHB starch was an alternative source integrated into BHB cookie products.

### 3.7 Solubility and swelling power

**Figure 4:**
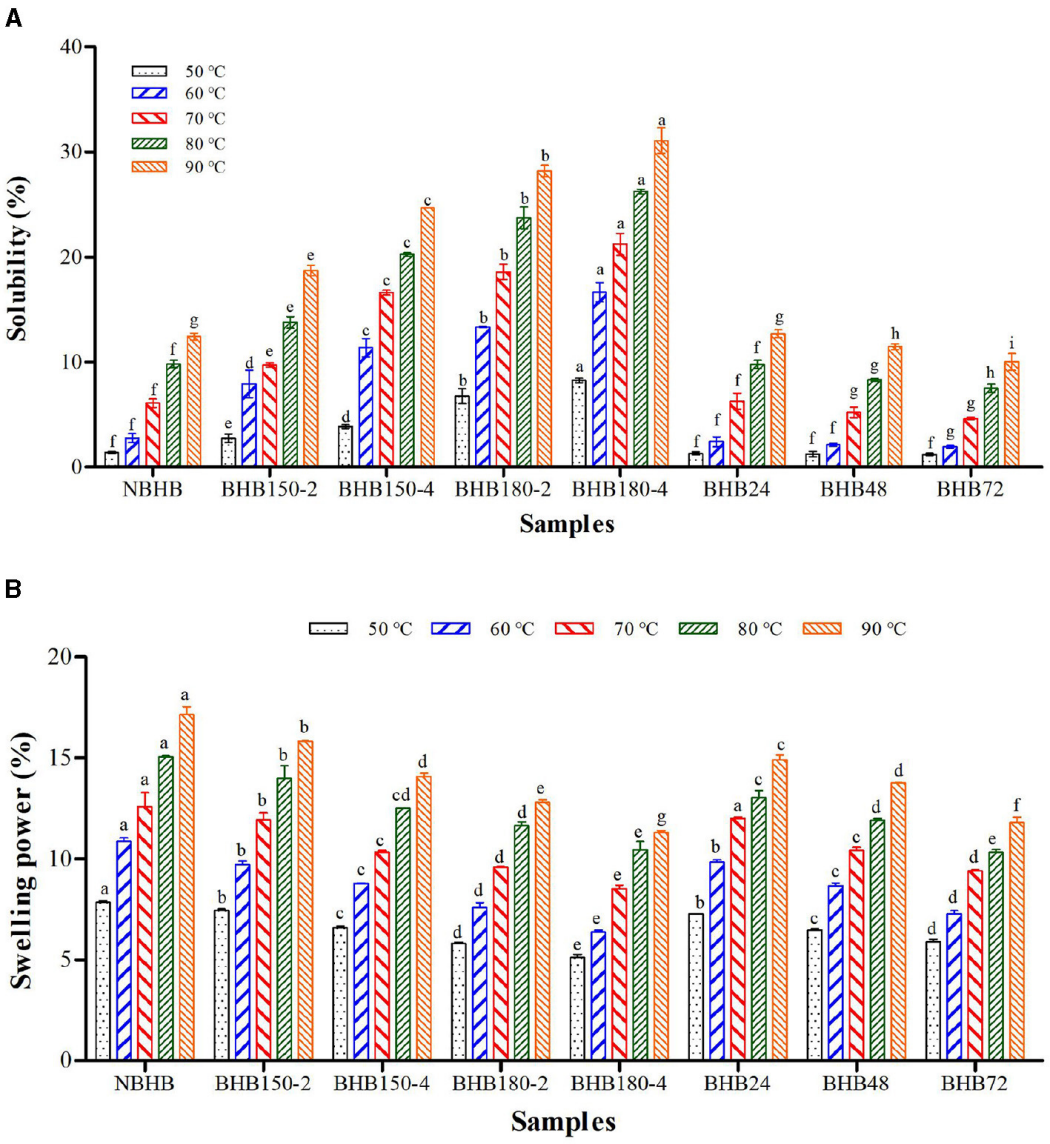
Solubility **(A)** and swelling power **(B)** of different samples. Bars bearing different letter within the same temperature are significantly different (*p* < 0.05).

The effect of temperature on solubility and SP of different samples is presented in Figure 4. Although significant differences in both solubility and SP were observed among the native and modified starches, they had opposite change trends for DHT- and ANN-modified samples, respectively. The solubility and SP of samples significantly raised with test temperature increasing. Compared to that of NBHB, the solubility of DHT-modified starches significantly increased (Figure 4A), while the SP decreased (Figure 4B). Due to high temperature conditions, partial double helices in NBHB began to be fractured into short molecules during DHT, which induced a loosening even collapsing granule structure. The high amount of short-chain amylose could easily diffuse out of granule and be dissolved to raise the solubility. These results were in good agreement with the finding in Section 3.6. Furthermore, the interactions among starch chains, double helices transition and melting of crystallites might be other factors to increase the solubility after DHT (2). However, the ANN-modified samples had significantly lower solubility than that of NBHB at each test temperature, and this decrease was positively connected with treatment time prolonging. During ANN, the restricted starch hydration, which resulted from higher degree of crystalline perfection and more rearrangement of starch molecules, was the main reason for the solubility reduction. Moreover, the interactions among starch chains and strengthening of bonds generated more stable structures. These further limited the migration and leaching of amylose, ultimately inducing lower solubility.

Both DHT and ANN could significantly decrease the SP of NBHB, and this decrement was positively related to temperature and duration during DHT as well as to treatment time for ANN. The gradual decrease in SP of DHT-modified starches was ascribed to the combination of starch molecules based on the amylose–amylopectin interaction during DHT (33). Meanwhile, this SP decrease was due to hindered diffusion of amylopectin molecules after rearranging the crystalline regions. For ANN-modified samples, the structural changes in granules, such as higher crystallinity, stronger molecular organization and more bonding force of small networks, contributed to the decrease in SP. The changes in amylopectin structure and AMC were other factors causing the SP reduction of ANN-modified starches. In addition, Tester and Morrison (44) reported that the formation of some new crystallites and amylose-lipid complexes after ANN improved the granule stability to decrease the SP. These results identified that both DHT and ANN limited the SP of NBHB under current circumstances, while DHT had contradictory influence on solubility.

### 3.8 DSC

The gelatinization parameters (*T*_o_, *T*_p_, *T*_c_, Δ*H*, etc.) of native and modified samples are shown in Table 3. Compared to those of NBHB, the *T*_o_, *T*_p_, *T*_c_ and Δ*H* of DHT-modified starches significantly decreased along with treatment temperature and duration increasing, whereas ANN induced higher values of these gelatinization parameters. Consistent effects of DHT and ANN on gelatinization properties of different starches had been respectively reported (2, 19).

**Table 3:** Gelatinization characteristics of native and modified starches.

**Samples**	***T*_o_ (°C)**	***T*_p_ (°C)**	***T*_c_ (°C)**	***T*_c_ – *T*_o_ (°C)**	**Δ*H* (J/g)**
NBHB	58.8 ± 0.33c	62.2 ± 0.18c	69.5 ± 0.75c	10.7 ± 0.42b	7.9 ± 0.03d
BHB150-2	57.2 ± 0.91d	59.9 ± 0.62d	67.4 ± 0.22d	10.2 ± 0.70b	7.4 ± 0.11e
BHB150-4	56.5 ± 0.13d	59.2 ± 0.24de	66.5 ± 0.05e	10.0 ± 0.08bc	6.9 ± 0.01e
BHB180-2	55.4 ± 0.21e	58.6 ± 0.21e	64.7 ± 0.28f	9.3 ± 0.07c	6.7 ± 0.06f
BHB180-4	55.1 ± 0.35e	57.5 ± 0.04f	63.5 ± 0.62g	8.4 ± 0.27d	6.2 ± 0.01g
BHB24	66.7 ± 0.06b	69.5 ± 0.17b	77.6 ± 0.11b	10.9 ± 0.06b	9.3 ± 0.01c
BHB48	70.0 ± 0.12a	72.4 ± 0.11a	81.1 ± 0.11a	11.1 ± 0.02a	10.1 ± 0.16b
BHB72	70.7 ± 0.47a	72.6 ± 0.29a	81.9 ± 0.05a	11.2 ± 0.41a	10.7 ± 0.03a

Means of triplicate determination ± SD with different letter in a column within each parameter are significantly different (p < 0.05).

T_o_, onset temperature; T_p_, peak temperature; T_c_, concluding temperature; T_c_-T_o_, gelatinization temperature range; ΔH, transition enthalpy.

In general, the gelatinization temperatures of starch represent the crystalline perfection, where higher *T*_o_ means more perfect crystallites in starch (45). Significant decrease in *T*_c_, *T*_o_ and *T*_p_ of DHT-modified samples revealed the inhomogeneity of double helix crystallites increased, and this was in accordance with the results from the XRD analysis. Different from DHT, the ANN could reinforce helical packing of amylopectin to reduce amorphous area, which also led to the formation of new double helices, and these contributed to higher *T*_o_, *T*_p_, and *T*_c_ values together. Moreover, the improved perfection of preexistent crystallites and crystalline structure in granules during ANN also increased the *T*_o_, *T*_p_, and *T*_c_. The starch chain interactions suppressed granule swelling to further delay the gelatinization of ANN-modified samples (17).

The Δ*H* represents the double helix content and order of crystalline in starch (2). The Δ*H* value of DHT-modified starches gradually decreased from 7.9 J/g (NBHB) to 6.2 J/g (BHB180-4) with treatment temperature and during raising. This shift was mainly ascribed to the amylose disruption. The destruction of concentrated crystalline region after DHT (shown in Figure 2), which further reduced ordered structure, induced the decrement in gelatinization temperatures and Δ*H*. However, the elevated Δ*H* of ANN-modified starches was based on the increased order degree of double helices, which was also supported by the results from XRD analysis. Additional factors for increasing Δ*H* might include perfection of crystallites, interactions among starch chain and organization of crystalline region (19). Thus, the ANN-modified samples required more energy to be gelatinized. Similar effects of DHT and ANN on starch gelatinization had respectively been reported on waxy corn and high-amylose rice starches (25, 27), and on cassava starch (46).

### 3.9 RVA

The viscosity of starch, based on the friction among starch chains during gelatinization, affects its applicability and functionality in food industry. The RVA parameters of native and modified samples are summarized in Table 4. Compared to NBHB, the modified samples showed significantly decreased PV, SB, BD, and FV values, and DHT-modified starches had decreased PT while ANN-modified samples were with higher ones. These changes were positively related to temperature and duration among DHT-modified samples, as well as to treatment time for ANN-modified starches, with BHB180-4 having lowest viscosities and BHB72 having highest PT. These observations were in agreement with differential scanning calorimetry (DSC) analysis.

**Table 4:** Pasting properties of different samples.

**Properties**	**Samples**							
	**NBHB**	**BHB150-2**	**BHB150-4**	**BHB180-2**	**BHB180-4**	**BHB24**	**BHB48**	**BHB72**
PV (cP)	3,689.0 ± 9.8a	2,828.5 ± 8.6b	2,407.0 ± 6.3d	705.0 ± 4.2f	587.0 ± 11.3g	2,805.5 ± 0.7b	2,666.0 ± 4.1c	1,022.5 ± 13.4e
BD (cP)	2,106.0 ± 6.4a	1,907.0 ± 5.1b	1,639.0 ± 7.9c	200.0 ± 2.8f	106.5 ± 4.9g	1,209.0 ± 14.1d	589.5 ± 16.2e	126.0 ± 3.6fg
SB (cP)	1,775.5 ± 2.5a	1,585.0 ± 2.1c	1,475.0 ± 9.1d	372.5 ± 6.4f	305.5 ± 12.0g	1,647.5 ± 9.1b	1,088.5 ± 4.7e	419.5 ± 2.1f
FV (cP)	3,358.5 ± 5.5a	2,506.5 ± 4.3d	2,243.0 ± 9.5e	877.5 ± 7.8g	786.0 ± 8.3h	3,244.0 ± 2.4b	3,165.0 ± 7.1c	1,316.0 ± 11.3f
PT (°C)	75.4 ± 0.6d	74.6 ± 0.2e	73.4 ± 0.5f	72.9 ± 0.1f	71.1 ± 0.7g	81.5 ± 0.2c	87.3 ± 0.1b	89.7 ± 2.3a
Pt (min)	4.6 ± 0.1c	4.6 ± 0.2c	4.4 ± 0.3d	4.3 ± 0.1de	4.2 ± 0.1e	5.3 ± 0.1b	5.8 ± 0.1a	6.0 ± 1.2a

Means of triplicate determination ± SD with different letter in the row within each property are significantly different (p < 0.05). “cP” is the rapid viscosity units.

PV, peak viscosity; BD, breakdown viscosity; FV, final viscosity; SB, setback viscosity; PT, pasting temperature; Pt, Peak time.

Generally speaking, starch viscosity is mainly dependant on the source, size of granule, ratio of amylose to amylopectin, crystal structure, etc. The decrease in PV, SB, BD and FV of DHT-modified samples was mainly due to the thermal degradation of amylose, amylopectin and crystalline structure (25). The PV is associated with the maximum swelling capacity just before granule disintegration, which represents the SP of starch (16). The enhanced association among starch chains and improved intermolecular interaction in starch granules during DHT significantly decreased the PV with treatment temperature and duration increasing. This was consistent with the results from SP testing. The markedly decreased FV of DHT-modified starches was ascribed to changes in crystalline structure and short amylopectin. Significantly reduced SB suggested DHT improved the stability of cold starch paste under shear force. The decrease in both FV and SB identified a lower retrogradation trend of DHT-modified starches. Moreover, the BD of DHT-modified samples positively decreased with treatment temperature and duration raising. The reduction in both Pt and PT identified DHT-modified samples were gelatinized easier compared to NBHB. Unlike DHT, the ANN significantly decreased amylose leaching, increased crystallinity (Figure 2), promoted more interactions among starch chains and limited the SP (Figure 4B), which further decreased the PV and SB. The reduced SB and BD showed ANN could increase the heat resistance of ANN-modified samples, improving their stability during continuous heating and shearing, and this would extend the application (19). Meanwhile, the improved bond strength in ANN-modified starches needed higher energy to be broken. It increased the PT and delayed their Pt. These results showed that both DHT and ANN could significantly decrease the viscosity properties of BHB starch, but they had opposite effects on Pt and PT.

### 3.10 *In vitro* digestibility

**Figure 5:**
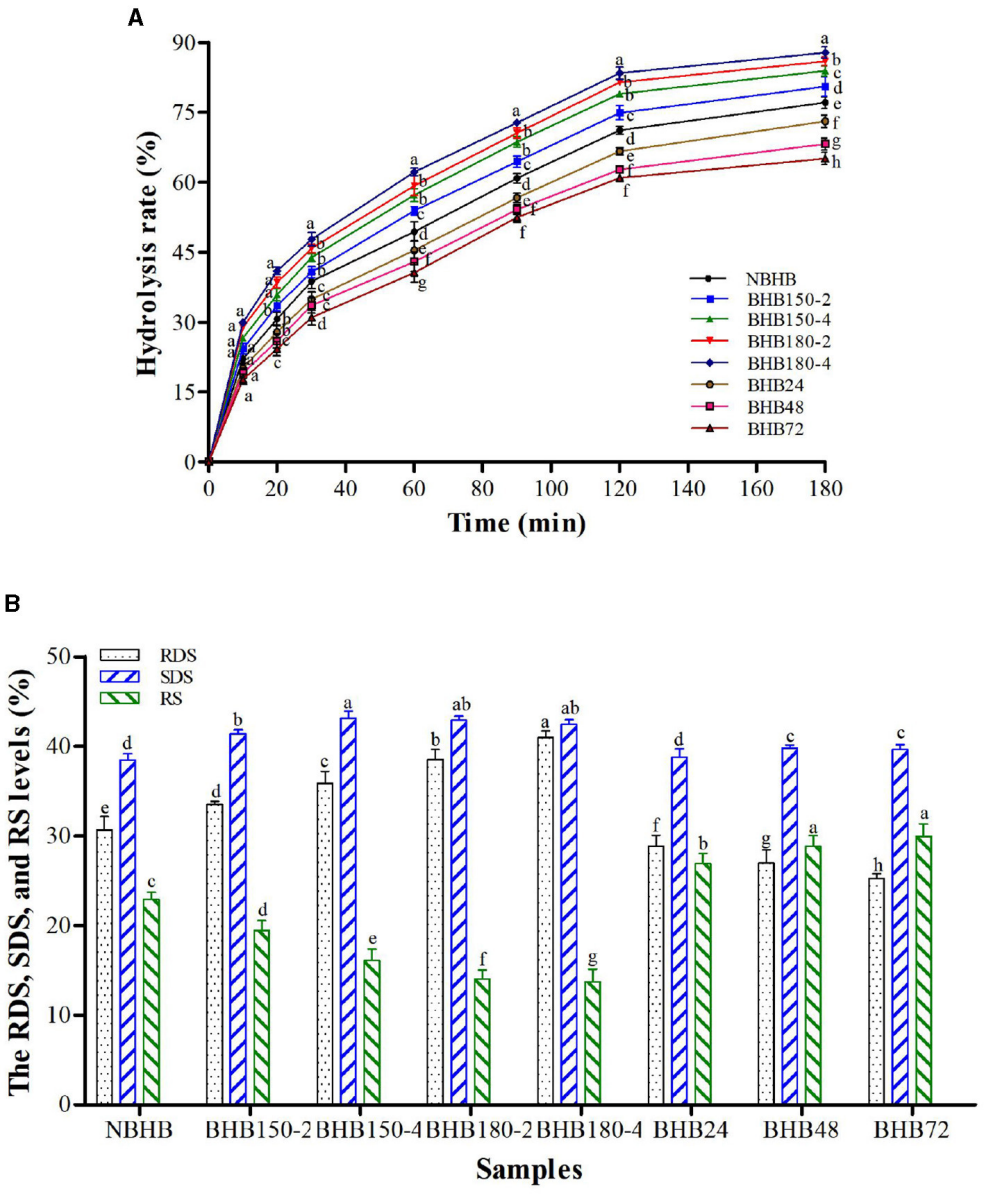
Hydrolysis rate **(A)** and RDS, SDS, and RS levels **(B)** of different samples. Different letters in same time point **(A)** and different samples **(B)** within same the property represent significant differences (*p* < 0.05).

The influence of both DHT and ANN on *in vitro* hydrolysis rate of BHB starch is presented in Figure 5A, and the level of RDS, SDS and RS in different samples is shown in Figure 5B. The hydrolysis rate of native and modified starches raised with digestion time prolonging, in the first 20 min of which, it increased sharply and gradually from 30 to 180 min. In comparison to NBHB, DHT-modified samples had significantly higher hydrolysis rate, while ANN-modified starches with lower values. After DHT, the RDS content significantly increased to reach a maximum value in BHB180-4 (40.96%), and the level of SDS and RS decreased. Opposite to DHT, the RDS content of starch was remarkably decreased by ANN, while the SDS and RS levels increased. These alterations of both DHT- and ANN-modified samples were positively dependant on individual treatment conditions. These results indicated that DHT significantly improved the *in vitro* digestibility of BHB starch, whereas ANN deceased. Similar changing trends had been previously found on waxy potato starch following DHT (47) and buckwheat starch after ANN (19).

The high temperature and low moisture during DHT promoted BHB starch granules with porous structure (Figure 1), which could draw digestive enzymes into the interior of granules to increase hydrolysis rate. The decreased AMC contributed DHT-modified starches more susceptible to enzymatic attack during digestion (48), further raising the RDS content. Additionally, the decrease in gelatinization temperatures and Δ*H* (Table 3) suggested the disintegration of double helices in crystalline region following DHT, and this increased the RDS level as well. Partial disruption or damage of organized starch chains and weak associations among starch molecules during DHT increased the susceptibility of BHB starch to enzyme, which was responsible to decrease RS content (29). During DHT, the transition from RS to RDS might account for these results as well. Generally, the AMC and crystalline structure are the most important factors influencing enzymatic susceptibility of ANN-modified samples (16). As shown in Table 2 and Figure 2, the ANN-modified samples had higher AMC and better crystalline perfection with higher crystallinity than those of NBHB. This mainly contributed lower hydrolysis rates and RDS content. The strong interactions among starch chains during ANN, partially restricted the accessibility of ANN-modified starch molecules to the enzymes, which raised the level of SDS and RS. These results suggested that ANN could decrease *in vitro* digestibility of BHB starch and improve its health benefits by reducing the RDS level and increasing the SDS and RS contents, whereas DHT had opposite effect.

### 3.11 PCA of the properties

**Figure 6:**
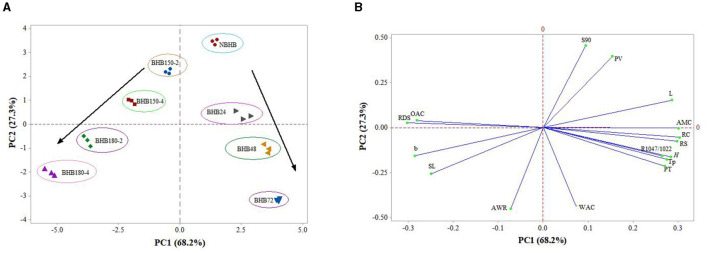
PCA plots summarizing the association among samples and their structural, physicochemical, and digestibility properties. **(A)** Clusters of different samples on the score plot; **(B)** PCA loading plot of different properties. OAC, oil absorption capacity; RDS, rapidly digestible starch; b, yellow/blue axis of color analysis; SL, solubility at 90°C; AWR, alkaline water retention; S90, solubility at 90°C; PV, peak viscosity; L, the lightness of samples; AMC, amylose content; RC, relative crystallinity; RS, resistant starch; *R*_1, 047/1, 022_, the absorbance ratio between 1,047 and 1,022 cm^−1^ in FT-IR; *H*, the enthalpy; Tp, peak temperature of DSC; PT, pasting temperature of RVA; WAC, water absorption capacity.

The PCA is a method for multivariate analysis, and it transforms original variables into new ones named principal components (PCs). The PCs can statistically represent data with biplot containing score and loading plots. For this study, the PCA was applied to show the interrelationships among structural, physicochemical, and *in vitro* digestive properties of native and modified starches in Figure 6. The PC1 and PC2 respectively represented 68.2 and 27.3%, totally explaining 95.5% of data variation.

Figure 6A showed eight groups that were representative of the separation trend by DHT and ANN with different treatment conditions. The NBHB and ANN-modified samples had positive scores on PC1, while DHT-modified samples were located at negative side. Meanwhile, eight groups were located at two different sides of PC2, and the distance between either two of them positively represented the degree of their discrepancy. Additionally, the DHT- and ANN-modified samples gradually moved further from NBHB to two different directions, identifying both DHT and ANN truly influenced the structural, physicochemical and *in vitro* digestive properties of BHB starch in different ways. The change in distance of DHT-modified samples was dependent on treatment temperature and duration, and the effect degree of ANN on samples was based on treatment time. The PCA was useful for predicting results of research on starch modification by DHT or ANN in the future.

The loading plot provided the correlation among structural, physicochemical and *in vitro* digestive properties of native and modified samples, which is shown in Figure 6B. Generally, the clusters formed by different properties show positive correlation among them, whereas negative relation to other clusters. The OAC, RDS, b, SL and AWR were loaded negatively on PC1, which were main contributors corresponding to DHT-modified samples on the score plot (Figure 6A). The main contributors corresponding to NBHB and ANN-modified were S90, PV, L, AMC, RC, RS, R1047/1022, *H*, Tp, PT, and WAC, which were positively located on PC1. Among these properties, the AMC was almost located on PC2 line. The S90, PV and L were positively correlated to each other, whereas negatively related with RC, RS, R1047/1022, *H*, Tp, PT, and WAC. Thus, these above PCA results identified that both DHT and ANN had marked influence on structural, physicochemical and *in vitro* digestive properties of BHB starch. But the influence from DHT and ANN were in two different ways and positively related to individual treatment conditions.

## 4 Conclusion

In this study, the effect of DHT and ANN on multi-structures, physicochemical properties and *in vitro* digestibility of BHB starch were measured. Compared to NBHB, the DHT and ANN-modified samples had significantly rougher morphology, higher AWR and water absorption capacity, as well as lower SP and viscosities. Both DHT and ANN did not change the “A”-type crystalline pattern and FT-IR spectroscopy of NBHB. The AMC, RC, L^*^ value, gelatinization parameters and PT were significantly reduced by DHT, while oil absorption capacity, a^*^ and b^*^ values increased, which were positively related to treatment temperature and time. However, for these properties, totally opposite alteration trends had been found among ANN-modified samples, which depended on treatment time during ANN. Furthermore, different influence on *in vitro* digestibility of NBHB were observed under DHT and ANN, respectively. Compared to those of NBHB, DHT markedly raised hydrolysis rate and RDS content, and decreased SDS ans RS levels mainly based on the degradation of starch chains, whereas ANN improved the hydrolyzation stability primarily because of interactions among starch molecules. Overall, these results revealed both DHT and ANN were effective technologies for NBHB modification, but they had distinguishing influence on properties with different underling mechanisms. That's why we have to select reasonable method to modify starch for different application purposes. Additionally, the ANN-modified BHB starch might be applied in noodles, infant and canned foods, while DHT-modified sample might develop functional food by further interacting with dietary polyphenols and lipids in the future.

## Data availability statement

The original contributions presented in the study are included in the article/supplementary material, further inquiries can be directed to the corresponding author.

## Author contributions

HL: Funding acquisition, Writing – original draft, Investigation, Writing – review & editing. SG: Formal analysis, Methodology, Conceptualization, Writing – original draft. GT: Resources, Writing – review & editing, Investigation. SZ: Methodology, Formal analysis, Writing – original draft. SL: Funding acquisition, Project administration, Supervision, Writing – review & editing.
